# Effect of roux-en Y gastric bypass surgery on patients with type 2 diabetes mellitus: A protocol of systematic review and meta-analysis

**DOI:** 10.1097/MD.0000000000020382

**Published:** 2020-06-05

**Authors:** Ke-Yan Chen, Ying-Li Liu, Jin-Cai Shang, De-Wang Su, Rong-Rong Yao, De-Zhi Ke, Hao Tian

**Affiliations:** a Department of Endocrinology; b Department of General Surgery; c Department of Interventional Radiology, First Affiliated Hospital of Jiamusi University, Jiamusi, China.

**Keywords:** effect, roux-en Y gastric bypass surgery, type 2 diabetes mellitus

## Abstract

**Background:**

Previous studies have reported that roux-en Y gastric bypass surgery (RYGBS) can benefit patients with type 2 diabetes mellitus (T2DM). However, their conclusions are still inconsistent. Thus, this study will aim to assess the effect of RYGBS for patients with T2DM.

**Methods:**

In this study, the electronic databases of MEDLINE, EMBASE, CENTRAL, CINAHL, AMED, and CNKI from inceptions to the present without any limitations to language and publication status. All randomized controlled trials on assessing the effect of RYGBS for patients with T2DM will be included in this study. Two independent authors will carry out study search and selection according to the previous designed inclusion and exclusion criteria. At the same time, 2 authors will independently evaluate the risk of bias assessment by Cochrane risk of bias tool. Any disagreements between 2 authors will be solved by a third author through discussion. RevMan 5.3 software will be utilized for statistical analysis.

**Results:**

This study will summarize the most recent studies and will provide a deeper understanding about using the effect of RYGBS for patients with T2DM.

**Conclusions:**

The findings of this study will present the existing evidence for the effect of RYGBS for patients with T2DM.

**Systematic review registration:**

INPLASY202040127.

## 1 Introduction

Type 2 diabetes mellitus (T2DM) is one of the most common metabolic diseases.^[1–3]^ It occurs because of the hyperglycemia with disorders in the metabolism of proteins, lipids, and carbohydrates.^[4–6]^ It is estimated that about 422 million people experience diabetes mellitus, and more than 90% diabetes mellitus patients are T2DM.^[7–9]^ The etiology of T2DM involves obesity and insulin resistance.^[10–12]^ Thus, it is becoming more prevalence among obesity population.^[13–14]^ Previous studies have reported that roux-en Y gastric bypass surgery (RYGBS) has used to treat patients with T2DM.^[15–25]^ However, there is no literature evidence addressing on the effect of RYGBS for patients with T2DM at evidence-based medicine level. Thus, the target of this study is to evaluate the effect of RYGBS for patients with T2DM.

## 2 Methods

### 2.1 Objective

This study aims to evaluate the effect of RYGBS for patients with T2DM.

### 2.2 Study registration

This protocol review has been registered on INPLASY202040127. We have reported this study based on the Preferred Reporting Items for Systematic Reviews and Meta-Analyses Protocols guideline.

### 2.3 Inclusion criteria for study selection

#### 2.3.1 Types of studies

All randomized controlled trials (RCTs) on the effect of RYGBS for patients with T2DM will be included without any language or publication restrictions. However, any other studies, except RCTs will not be included.

#### 2.3.2 Types of patients

Participants who were clinically diagnosed as T2DM will be included without limitations of gender, age, economic status, and educational background.

#### 2.3.3 Types of interventions

In the experimental group, all participants received RYGBS intervention.

In the control group, all participants who received any other interventions, except RYGBS will be included.

#### 2.3.4 Types of outcome measurements

The primary outcomes include partial remission of T2DM (defined as blood HbA1c < 6.5% (48 mmol/mol)), and complete remission of T2DM (defined as blood HbA1c < 6% (42 mmol/mol)).

The secondary outcomes consist of hemoglobin A1c, body mass index, lipids, high sensitivity C-reactive protein, tumor necrosis factor-α, and high molecular weight adiponectin.

### 2.4 Search methods for the identification of studies

#### 2.4.1 Search electronic databases

In this study, we will search the following databases of MEDLINE, EMBASE, CENTRAL, CINAHL, AMED, and CNKI. All these databases will be searched from inceptions to the present. All those databases will be searched with no restrictions of language and publication status. We will include RCTs on evaluating the effect of RYGBS for patients with T2DM. We will build an example of detailed search strategy for MEDLINE in Table 1. We will adapt similar search strategies for other electronic databases.

**Table 1:**
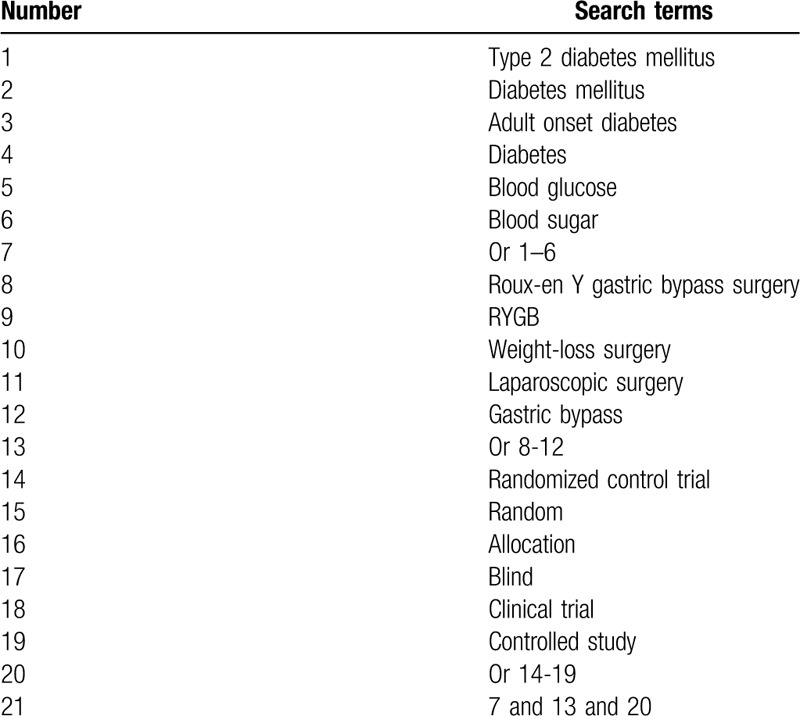
Search strategy applied in MEDLINE.

#### 2.4.2 Search for other resources

In addition, we will also search conference abstracts, dissertations, and reference lists of associated reviews.

### 2.5 Data collection

#### 2.5.1 Study selection

Two authors will independently scan titles/abstracts against the previous eligibility criteria. If any uncertainty exists, we will obtain and read full-texts for further clarification. We will record any reasons for all excluded studies. If there are diversities between 2 authors, a third author will be invited to solve these different views through discussion. The process of study selection will be shown in the flow diagram.

#### 2.5.2 Data extraction and management

Two authors will independently extract data from the included studies based on the standardized data collection form. Any different opinions between 2 authors will be solved and discussed by a third author. Extracted data consist of title, first author, publication year, study characteristics, patient characteristics, study setting, inclusion and exclusion criteria, diagnostic criteria, study design, details of interventions and controls, outcome assessments, safety, and funding information.

#### 2.5.3 Dealing with missing data

If there are any unclear or missing data, we will contact original authors to request that kind of data. If we cannot obtain such data, we will analyze the available data.

#### 2.5.4 Risk of bias in included studies

Two authors will independently assess the risk of bias using Cochrane Risk of Bias Tool, which was developed to assess the methodological quality. Using this tool, all risk of bias will be divided as low, unclear, and high risk of bias. Any divergences between 2 authors will be worked out by a third author through discussion.

### 2.6 Statistical analysis

#### 2.6.1 Data synthesis

We will use RevMan 5.3 software for statistical analysis in this study. We will present dichotomous data using risk ratio and 95% confidence intervals. We will show the continuous data using mean difference or standardized mean difference and 95% confidence intervals. We will utilize the *I*^2^ statistic test to identify heterogeneity among included studies. The value of *I*^2^ ≤ 50% means reasonable heterogeneity, and we will use a fixed-effect model. We will undertake meta-analysis if sufficient data on the similar characteristics of study and patient, interventions, controls, and outcomes are obtained. On the hand other, the value of *I*^2^ > 50% shows obvious heterogeneity, and we will utilize a random-effect model. Additionally, we will perform subgroup analysis to investigate any factor that may result in such significant heterogeneity.

#### 2.6.2 Unit of analysis

If there are any cross-over studies which were entered into this study, we will only analyze the data of first study period.

#### 2.6.3 Subgroup analysis

Subgroup analysis will be undertaken based on the different types of interventions and control therapies, and outcome assessments.

#### 2.6.4 Sensitivity analysis

Sensitivity analysis will be performed to explore the stability of the pooled outcome results by removing low quality studies.

#### 2.6.5 Reporting bias

If more than 10 eligible RCTs are included, we will conduct Funnel plot, Egger linear regression test and Begger rank correlation test to check if there are any potential reporting biases.^[26]^

### 2.7 Dissemination and ethics

This study will be disseminated on a peer-reviewed journal. It will not need ethic approval, because it will only use published data.

## 3 Discussion

Although a similar systematic review has been published several years ago, there is still a variety of high-quality studies that have been published after it. This study will summarize the up-to-date evidence to assess the effect of RYGBS for patients with T2DM. The results of this study will provide the evidence to judge whether RYGBS can benefit patients with T2DM. In addition, its results will yield helpful evidence for both clinical practice and further researches.

## Author contributions

**Conceptualization:** Ke-Yan Chen, Ying-Li Liu, De-Wang Su, Hao Tian.

**Data curation:** Ying-Li Liu, Jin-Cai Shang, Rong-Rong Yao, De-Zhi Ke, Hao Tian.

**Formal analysis:** Ke-Yan Chen, Jin-Cai Shang, De-Wang Su, Rong-Rong Yao, De-Zhi Ke.

**Investigation:** Jin-Cai Shang, Hao Tian.

**Methodology:** Ying-Li Liu, De-Wang Su, Rong-Rong Yao, De-Zhi Ke.

**Project administration:** Hao Tian.

**Resources:** Ke-Yan Chen, Ying-Li Liu, Jin-Cai Shang, De-Wang Su, Rong-Rong Yao, De-Zhi Ke.

**Software:** Ke-Yan Chen, Ying-Li Liu, Jin-Cai Shang, De-Wang Su, Rong-Rong Yao, De-Zhi Ke.

**Supervision:** Ying-Li Liu.

**Validation:** Ke-Yan Chen, Ying-Li Liu, Jin-Cai Shang, De-Wang Su, Rong-Rong Yao, De-Zhi Ke, Hao Tian.

**Visualization:** Ke-Yan Chen, Rong-Rong Yao, Hao Tian.

**Writing – original draft:** Ke-Yan Chen, Ying-Li Liu, Jin-Cai Shang, De-Wang Su, De-Zhi Ke, Hao Tian.

**Writing – review & editing:** Ke-Yan Chen, Jin-Cai Shang, De-Wang Su, Rong-Rong Yao, Hao Tian.
